# Comment on “Charge Transfer-Triggered Bi^3+^ Near-Infrared Emission in Y_2_Ti_2_O_7_ for Dual-Mode Temperature Sensing”

**DOI:** 10.1021/acsami.3c11758

**Published:** 2023-09-11

**Authors:** Hei-Yui Kai, Longbing Shang, Ka-Leung Wong, Chang-Kui Duan, Peter A. Tanner

**Affiliations:** † Department of Chemistry, Hong Kong Baptist University, Waterloo Road, Kowloon Tong 999077, Hong Kong S. A. R., P. R. China; ‡ CAS Key Laboratory of Microscale Magnetic Resonance, School of Physical Sciences, University of Science and Technology of China, Hefei 230026, P. R. China; § CAS Center for Excellence in Quantum Information and Quantum Physics, University of Science and Technology of China, Hefei 230026, P. R. China; ∥ Department of Applied Biology and Chemical Technology, The Hong Kong Polytechnic University, Hung Hom, Kowloon 999077, Hong Kong S. A. R., P. R. China

**Keywords:** Mn^4+^ emission, Cr^3+^ emission, Fe^3+^ emission, impurity emission, pyrochlore, Bi^3+^ doping

## Abstract

Undoped Y_2_Ti_2_O_7_ exhibits impurity emission bands at low temperatures due to Mn^4+^ and Cr^3+^, as established by codoping with these ions. Contrary to a recent report by Wang et al., *ACS Appl. Mater. Interfaces***2022**, *14*, 36834–36844, we do not observe Bi^3+^ emission in this codoped host, as also is the case for Fe^3+^. The emission reported in that paper as being due to Bi^3+^ in fact corresponds to Cr^3+^ emission. The Cr^3+^ and Mn^4+^ emissions are quenched with increasing temperature, so that Mn^4+^ emission is scarcely observed above 80 K. We present variable temperature optical data for Y_2_Ti_2_O_7_ and this host codoped with Mn, Cr, Fe, and Bi, as well as a theoretical justification of our results.

## 1 Introduction

**Figure 1:**
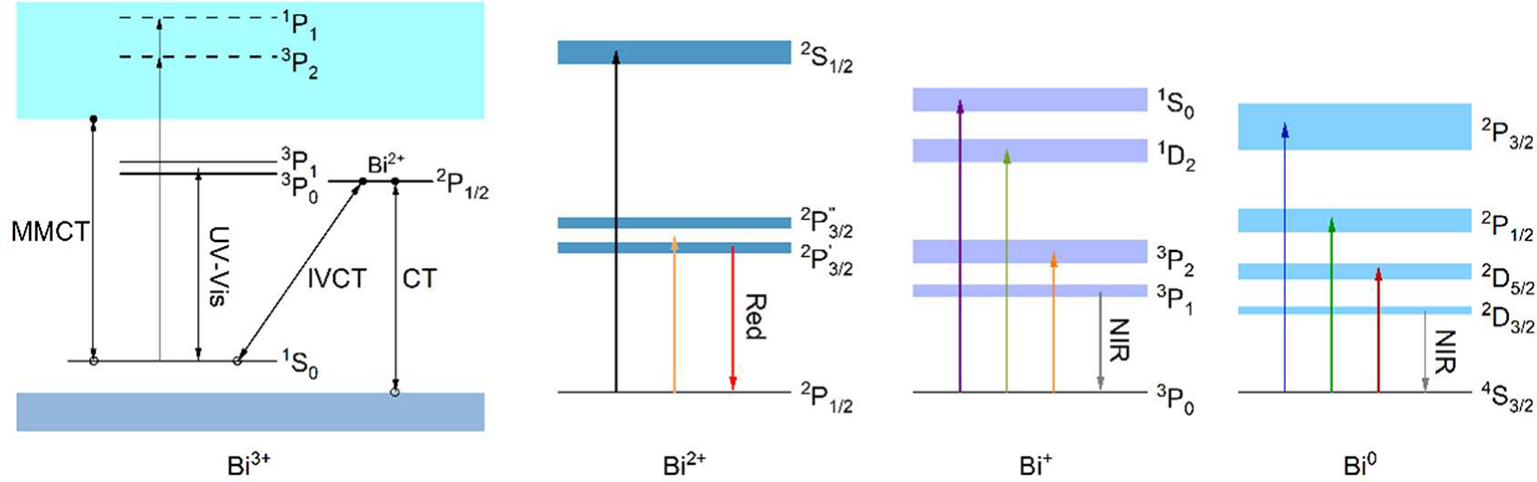
Some of the possible emission transitions of bismuth in undoped or doped compounds. MMCT, IVCT, and CT, respectively, represent Bi^3+^ to host cation ion (Ti^4+^ in Y_2_Ti_2_O_7_) termed metal to metal, Bi^3+^ to Bi^3+^ intervalent, and ligand to Bi^3+^ charge transfer transitions, which involve the transfer of an electron from the former (denoted as an open circle) to the latter (denoted as a filled circle). The emission of Bi^3+^ can be ^3^P_0,1_ to ^1^S_0_, CT, or MMCT, depending on which of these three sets of excited states is lower in energy. For Bi^2+^, ^2^P_3/2_^′^ and ^2^P_3/2_^′′^ represent the two crystal field levels of ^2^P_3/2_. UV–vis, Red, and NIR represent ultraviolet to visible, red, and near-infrared emissions.

Bismuth luminescence has recently attracted much attention, both theoretically^1−6^ and experimentally,^7−12^ because of potential applications as phosphors for light-emitting diodes, quantum-cutting materials suitable for silicon solar cells, and ratiometric temperature sensors.^13^ It is an attractive and versatile phosphor because its emission can occur in the ultraviolet (UV), green, red, near-infrared, and infrared spectral regions, depending upon its oxidation state^14^ (Figure 1).

The emission of Bi^3+^ has been reported in many oxide systems, including oxides,^5,15^ aluminates,^2,16,17^ zirconates,^18^ germanates,^19,20^ silicates,^21^ scandates,^22^ vanadates,^23^ titanates,^24^ and phosphates,^25,26^ and it is a coactor in many of these systems. Historically, Bi^3+^ has been known as a near UV-emitter, from ^3^P_1_,^3^P_0_ states to the ^1^S_0_ ground state, usually in the region of 300–400 nm, although the host lattice can modify the emission wavelength.^27^ The Stokes shift of Bi^3+^ is relatively small: ∼30 nm in Cs_2_NaBiCl_6_,^14^ ∼60 nm in Bi^3+^-doped LiScGeO_4_,^28^ and 38 nm for Ca_10_P_6_O_25_ doped with Bi^3+^.^29^ Blasse and Bril^30^ also recognized that Bi^3+^ could be a universal sensitizer. Unlike Bi^2+^, it is not toxic, and systems doped with it are more easily synthesized. A Scopus search on May 2, 2023, showed that only 166 documents published between 1966 and 2000 concerned the emission of Bi^3+^. However, now the total is 1964 papers, up to 2023, showing the surge of interest in this sensitizer/activator.

We were therefore surprised to encounter a publication concerning room temperature Bi^3+^ emission in Y_2_Ti_2_O_7_ at 744 nm (lifetime ∼17 μs), with the Stokes shift of 353 nm.^12^ The emission band sharpens at 77 K with a maximum at 737 nm. Besides, excitation bands at 485 and 645 nm were reported in addition to the dominant band at 391 nm. These do not correspond to Bi^3+^ energy levels. Overall, we did not find the explanation of the emission band as Bi^3+^–Ti^4+^ metal–metal charge transfer to be convincing. The intensity of the emission band was reported to decrease for Bi^3+^ concentrations >0.5%.

Srivastava et al. have summarized the low temperature optical properties of Y_2_Ti_2_O_7_.^31^ The broad band emission, due to O^2–^ (2p) → Ti^4+^ (3d^0^) charge transfer (CT), peaks at 485 nm. The corresponding excitation peak is at 310 nm. These wavelengths are in agreement with those from the earlier study of Alarcon and Blasse.^32^ The incorporation of Bi^3+^ into the Y^3+^ site shifts both bands to longer wavelengths, so that the peak of the emission band is located at 550 nm for ∼5% Bi^3+^ doping.^31^ The excitation band was assigned to the Bi^3+^ (6s^2^) → Ti^4+^ (3d^0^) metal–metal CT transition.^31^ The CT emission band is quenched at low temperature: the intensity decreases by a factor of 8–9 from 10 to 150 K. These phenomena are analogous to the metal–metal CT bands assigned in YVO_4_:Bi^3+^^33^ and La_2_Zr_2_O_7_:Bi^3+^.^34^ It therefore appears that the previous results for Y_2_Ti_2_O_7_:Bi^3+^ call into question the more recent work of Wang et al.^12^

We are aware of the effects of minute amounts of impurity ions, notably transition metals, upon host luminescence.^2^ Notably, the luminescence of Cr^3+^-doped Y_2_Ti_2_O_7_ has been investigated both experimentally^35^ and theoretically.^36,37^ Also, the optical spectra of Mn^4+^ in Y_2_Ti_2_O_7_ have been investigated at low temperature.^38,39^ In addition, Kushnikova et al.^40^ reported an intense broad absorption band at 505 nm, attributed to the ^5^E_g_ → ^5^T_2g_ transition of Mn^3+^ ions replacing Y^3+^.

The aim of the present study is to provide a valid assignment for the previously published luminescence of Y_2_Ti_2_O_7_, attributed to Bi^3+^ doping,^12^ and we therefore have a useful framework upon which to base our reinvestigation of the spectra of Y_2_Ti_2_O_7_ doped with tri- and tetra-positive ions.

## 2 Results and Discussion

### 2.1 Host Crystal Structure

**Figure 2:**
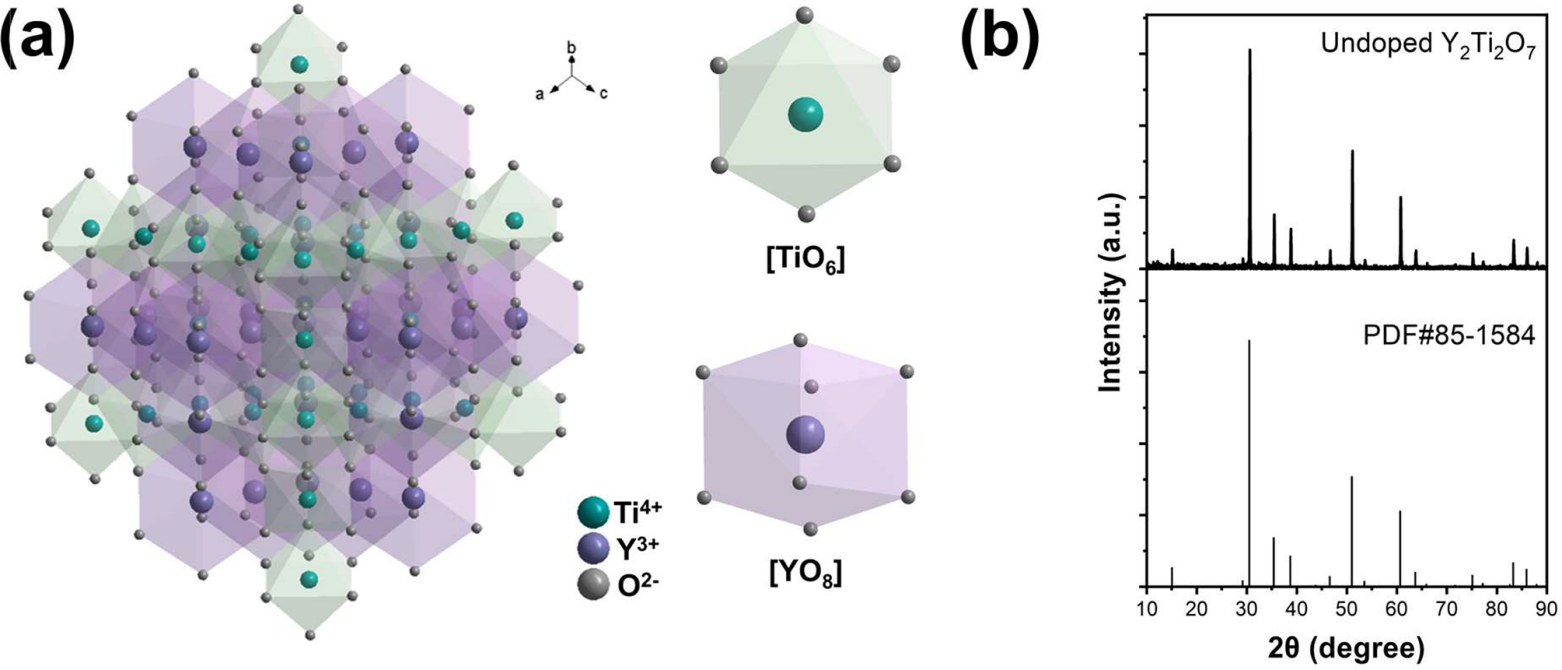
(a) Structure of Y_2_Ti_2_O_7_. (b) XRD trace of undoped sample and comparison with the standard card.

Crystals and powders of Y_2_Ti_2_O_7_ have been grown in previous studies by several methods, including microwave synthesis,^41^ the optical floating zone technique,^42,43^ and the straightforward solid state solution method,^12^ as employed herein (Section 4). The structure of Y_2_Ti_2_O_7_ has been described by Wang et al.^12^ It crystallizes in space group *Fd*3*m* (No. 227), *Z* = 8, with YO_8_ and TiO_6_ units interconnected by shared oxygens. Each cation has D_3d_ site symmetry. A sketch of the structure is made in Figure 2a, and our XRD results are compared with the standard card in Figure 2b.

### 2.2 Optical Properties of Undoped Material

**Figure 3:**
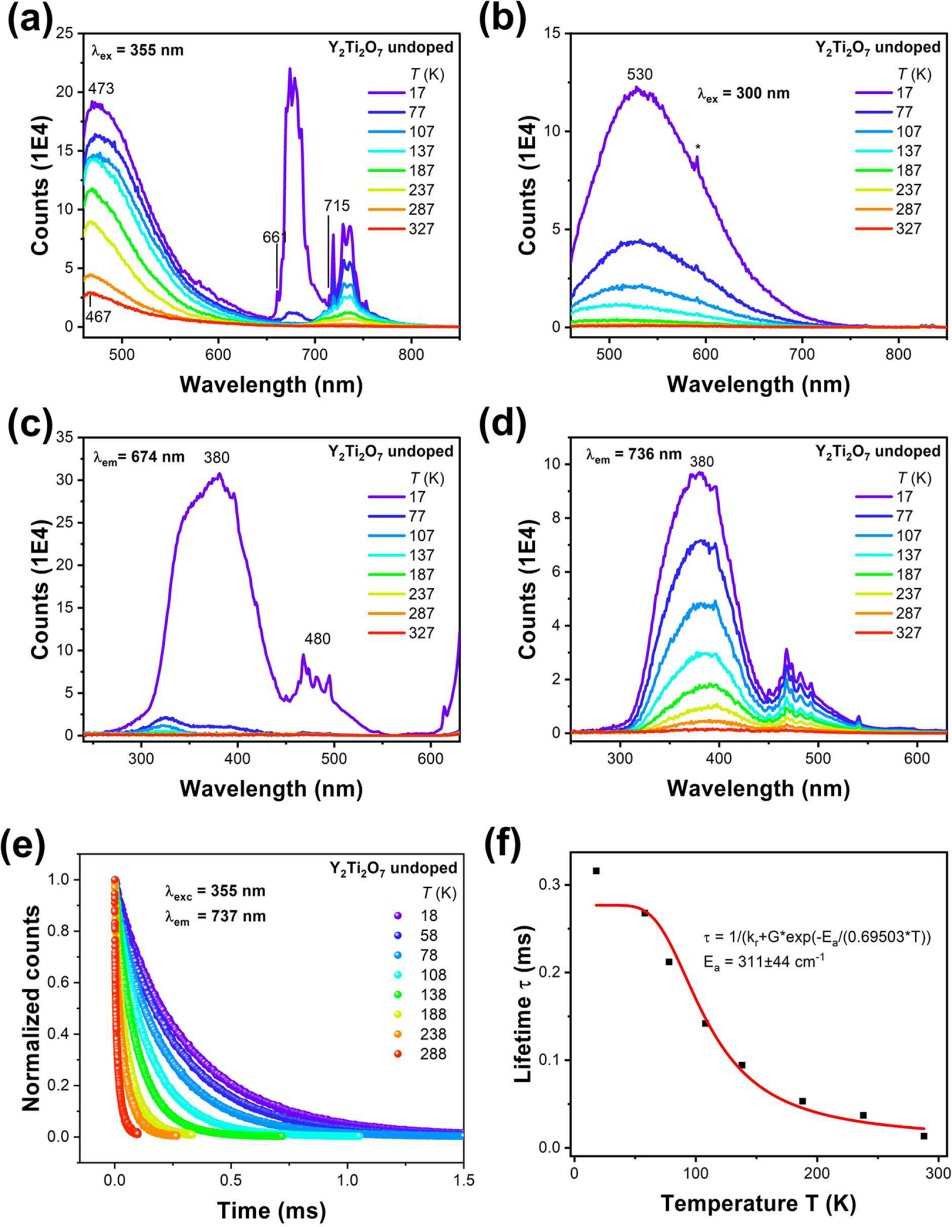
Variable temperature emission spectra of Y_2_Ti_2_O_7_ under (a) 355 nm and (b) 300 nm excitation. Excitation spectra of the (c) 674 nm and (d) 736 nm emission spectra. The sharp features at ∼480 nm here and in subsequent spectra are Xe lamp lines. (e) Decay curves of 737 nm emission using 355 nm excitation, from 18 to 288 K. (f) Single barrier model fit to monoexponential lifetimes determined from panel (e).

Figure 3a shows the 355 nm excited emission spectrum between 17 and 327 K of undoped Y_2_Ti_2_O_7_ prepared from 99.995% TiO_2_ and 99.999% Y_2_O_3_. The spectra recorded using 375 and 395 nm excitation are similar, but when normalizing at 473 nm, the relative intensities of lower energy bands decrease with longer wavelength excitation (not shown). Three groups of bands are observed. The broad band at the shortest wavelength (∼470 nm in Figure 3a) under 355 nm excitation is almost quenched at room temperature. Upon shorter wavelength excitation, Figure 3b, a band is observed at 530 nm, similar to the broad band observed by Alarcon and Blasse under 300 nm excitation for Y_1.998_Eu_0.002_Ti_2_O_7_ at 4.2 K.^32^ This feature has previously been associated with O^2–^–Ti^4+^ CT.^31,32^ We make preliminary assignments of the ∼470 and 530 nm bands. According to the formation energies, there is only a very small probability of Y–Ti antisites. Hence, the first of these bands is associated with unperturbed (Ti^3+^, O^–^) to (Ti^4+^, O^2–^) CT emission and the latter to the scenario of perturbed CT emission involving either Ti^4+^ near an oxygen vacancy or O^2–^ near a cation (Y^3+^) vacancy. The large breadth, excitation wavelength dependence, and low temperature quenching are therefore not unexpected.

The sharper structure beginning at 661 nm is quenched completely at room temperature. Third, the structured group of bands beginning at 714 nm becomes a broad band at room temperature. The 77 K spectrum in Figure 3a is very similar to our digitized 78 K emission spectrum of Figure 2a in ref (12), to wavelengths longer than 714 nm. The 237 K spectrum is also similar to the 298 K emission spectrum in the same figure. Our photomultiplier and grating setup has a cutoff after 730 nm, whereas the range of the Hamamatsu R928P photomultiplier tube employed in the equipment of ref (12) is from 180 to 920 nm. This accounts for the extension of bands to longer wavelength in the spectra of ref (12).

The corresponding excitation spectra are shown in Figure 3c and d and are quite similar for the 674 and 736 nm emission bands. The excitation spectrum of 520 nm emission (not shown) has a prominent band at 312 nm.

The lifetime of the 674 nm emission decreases from 0.48 ms at 18 K to 0.28 ms at 58 K. The lifetime of the emission at 737 nm can be followed to more elevated temperatures, and the recorded curves are shown and fitted in Figure 3e to give lifetimes in the millisecond or subms range. Fitting these lifetimes to a single barrier model (Figure 3f) indicates an activation energy in the region of 270–350 cm^–1^. Note that the lifetime of the bands at 719 and 737 nm at 288 K of our undoped sample is measured as 13 μs. The corresponding lifetime in Figure 2b of ref (12), attributed to Bi^3+^ emission, is 17 μs.

Our undoped sample of Y_2_Ti_2_O_7_ therefore contains impurity bands from species present in the starting materials or acquired during synthesis. The comparison with information presented in the Introduction clearly shows that the group of bands at shorter wavelengths of 715 nm corresponds to Mn^4+^ emission, whereas bands at longer wavelengths are due to Cr^3+^. We therefore synthesized samples of Y_2_Ti_2_O_7_ doped with these materials to confirm our suspicions.

### 2.3 Optical Properties of Mn-Doped Y_2_Ti_2_O_7_

**Figure 4:**
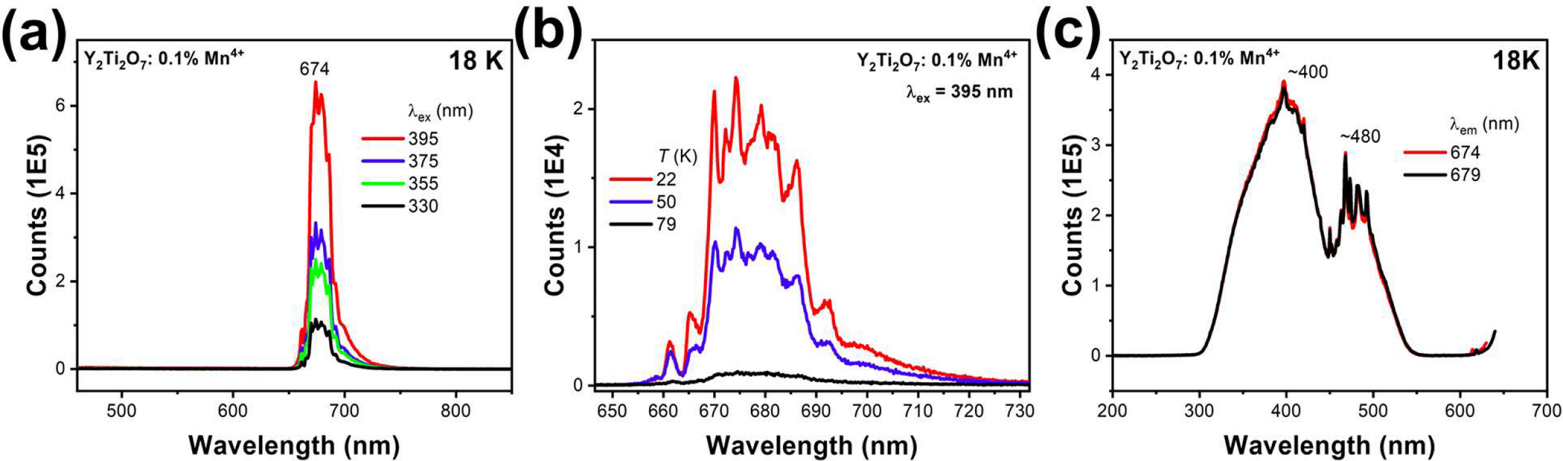
(a) Emission spectrum of Y_2_Ti_2_O_7_ doped with 0.1% Mn at 18 K using various excitation lines. (b) Temperature dependence of higher resolution spectrum and (c) corresponding excitation spectrum of panel (a).

Figure 4a shows the 18 K spectrum of 1% Mn-doped Y_2_Ti_2_O_7_ by using different Xe excitation lines of the Fluorolog instrument. Only one group of bands is observed, between 660 and 700 nm, corresponding to the ^2^E (^2^G) → ^4^A_2_ (^4^F) transition. The lifetime is 0.24 ± 0.03 ms, which is shorter than in undoped Y_2_Ti_2_O_7_. We are unable to assign the spectrum under higher resolution (Figure 4b) because the emission is almost quenched at 80 K. Possible reasons for the quenching are transfer to quenching centers or nonradiative relaxation to the electronic ground state. If the ZPL is at 661 nm, one would expect to observe hot bands at 79 K; otherwise, if the ZPL is assigned to the sharper band at ∼670 nm, then the Stokes and anti-Stokes vibrational energies do not match. The presence of several Mn^4+^ sites could be implied. Figure 4c shows that broad excitation bands are observed at 398 nm (^4^A_2_ → ^2^T_2_ (^2^G), ^4^T_1_ (^4^F)) and 479 nm (^4^A_2_ → ^4^T_2_ (^4^F)).

### 2.4 Optical Properties of Cr-Doped Y_2_Ti_2_O_7_

**Figure 5:**
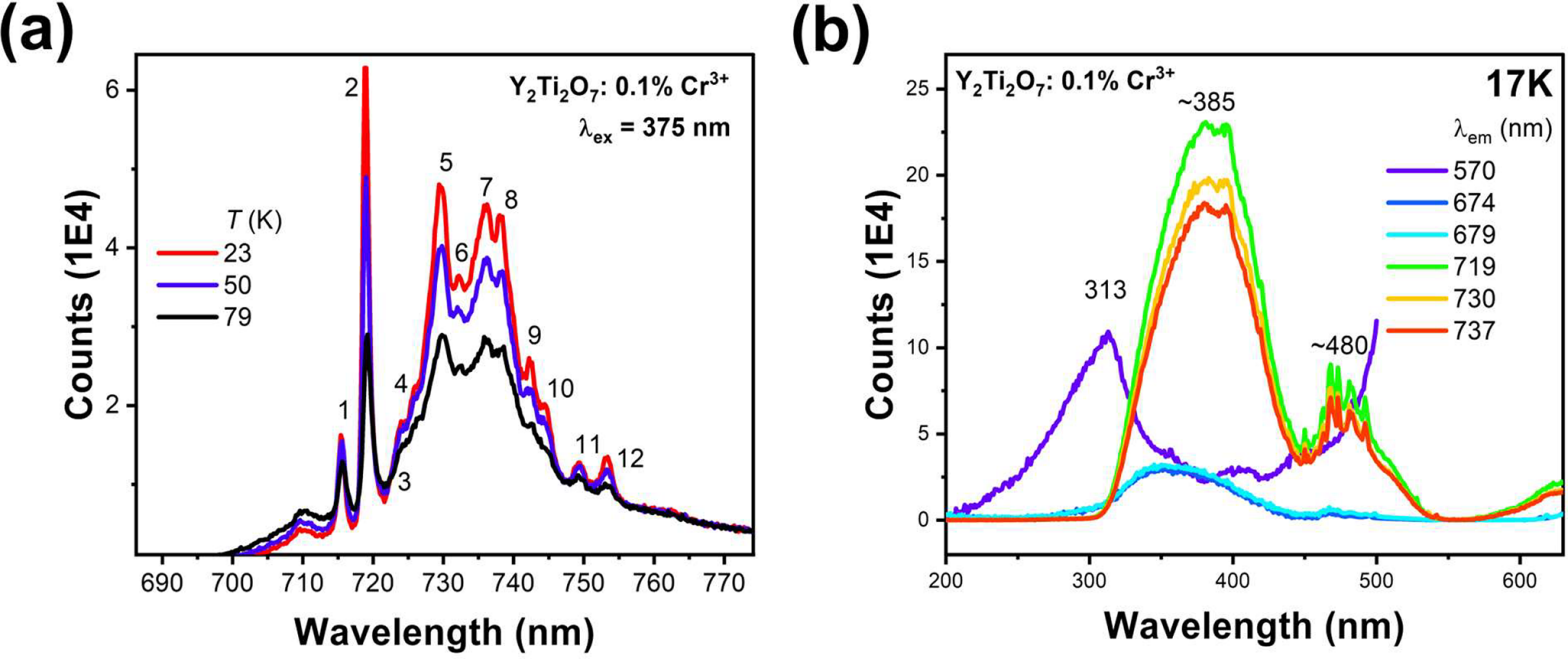
(a) Emission spectrum of Cr-doped Y_2_Ti_2_O_7_ at three temperatures as indicated. Refer to Table 1 for the line energies. (b) Excitation spectra of Cr-doped Y_2_Ti_2_O_7_ when monitoring different emission bands at 17 K.

The emission spectrum of Y_1.98_Cr_0.02_Ti_2_O_7_ is shown in Figure 5a. It is very similar to the 77 K spectrum of Cr-doped Y_2_Ti_2_O_7_ reported by Becker,^35^ with calibration differences between 14 and 33 cm^–1^ for the lines (denoted by Δ in Table 1). The D_3d_ double group site symmetry of Cr^3+^ spin–orbit levels in CrO_8_ or CrO_6_ leads to splitting of the excited states. Our calculation shows that Cr is unlikely to occupy the Y site due to high formation energy, even in the case of Y_2_O_3_ being depleted.

**Table 1:** Emission Spectra of Y_2_Ti_2_O_7_:Cr^3+^^a^

Line	Energy	Band locations	Δ	Energy	Assignment^35^
	14092	–115			
	14058	–81			
1	13977	E2 = 0	25	14002	E2; ^2^A(^2^E) → ^4^A_2_
2	13910	E1 67	20	13930	E1; E̅(^2^E) → ^4^A_2_
				13900	E2+E′ 102
3	13812	165	14	13826	E1+E′
4	13777	200	21	13798	E2+2E′
5	13708	269	16	13724	E1+2E′
6	13658	319	16	13674	E2+3E′+E′′
7	13586	391	15	13601	E1+3E′+E′′
8	13547	430	19	13566	E2+4E′+E′′
9	13470	507	22	13492	E1+4E′+E′′
10	13434	543	28	13462	E2+5E′+E′′
11	13347	630	33	13380	E1*+6E′
12	13277	700	30	13307	E2*+6E′

a The energies are in cm^–1^, and Δ represents the calibration difference of this work and ref (35). Refer to Figure 5a for line numbers. The stars in column 6 represent a typo in the original paper.

The two sharp bands (lines 1 and 2) are assigned to the Γ_4_, Γ_5,6_ (^2^E) → ^4^A_2_ zero phonon lines, separated by ∼70 cm^–1^. This splitting pattern is repeated upon the structure to lower energy. Becker has made spectral assignments in Table 1 which employ progressions in a mode of 102 cm^–1^ (E′), together with another vibration of 22 cm^–1^ (E′′). We do not concur with these assignments except to agree that lines 1 and 2 correspond to ZPL and that all of the remaining structure is vibronic in nature. The sparse data do not merit further discussion. The lifetime of the Cr^3+^ emission was measured as 0.25 ms at 17 K. The excitation spectra (Figure 5b) show bands at ∼385 and 480 nm when monitoring the Cr^3+^ emission. The Cr^3+^ emission is quenched for Cr^3+^ concentrations above 3%.

### 2.5 Optical Properties of Fe-Doped Y_2_Ti_2_O_7_

**Figure 6:**
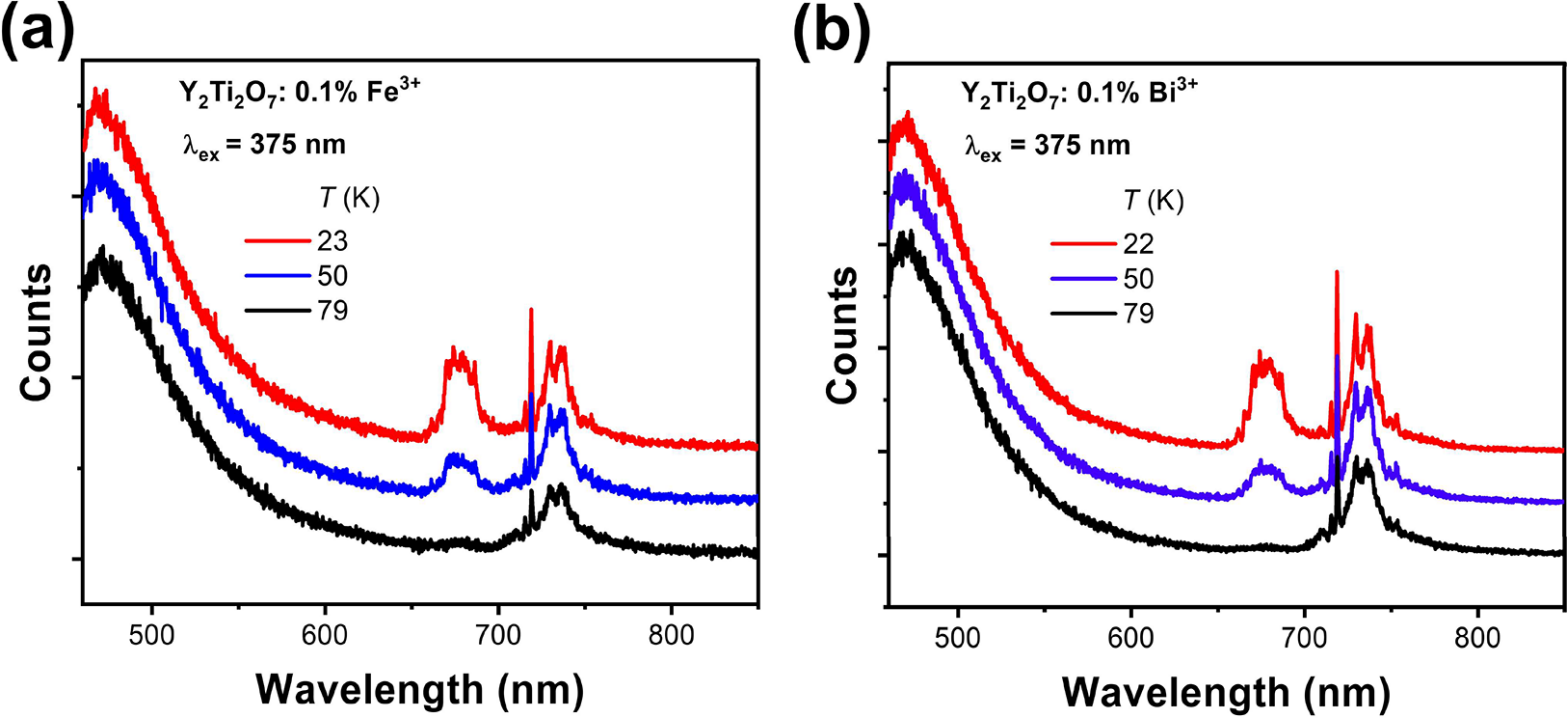
Emission spectra of (a) 0.1% Fe-doped Y_2_Ti_2_O_7_ and (b) 0.1% Bi-doped Y_2_Ti_2_O_7_ at selected temperatures.

Figure 6a shows the emission spectrum at three temperatures of 0.1% Fe-doped Y_2_Ti_2_O_7_. Bands due to Cr^3+^ and Mn^4+^ are observed, but there is no evidence of emission from Fe^3+^. Fe^3+^ ions normally act as quenchers of luminescence. Confirmed emissions of Fe^3+^ ions are from scenarios in which they occupy tetrahedral sites with a weak crystal field. There are no tetrahedral sites in Y_2_Ti_2_O_7_.

### 2.6 Optical Properties of Bi-Doped Y_2_Ti_2_O_7_

Figure 6b shows the emission spectrum of 0.1% Bi-doped Y_2_Ti_2_O_7_ at three temperatures. Just as in Section 2.5, there is no evidence of Bi^3+^ emission. The strong broad band at ∼470 nm corresponds to the Ti^4+^–O^2–^ CT transition. Bi^3+^ emission normally occurs in the near-UV spectral region, and the charge transfer band completely masks any other emission in this region. The intensity of the group of bands to a longer wavelength of 700 nm decreases markedly when Bi^3+^ is added to Y_2_Ti_2_O_7_.

### 2.7 Theoretical Rationalization of Results

**Figure 7:**
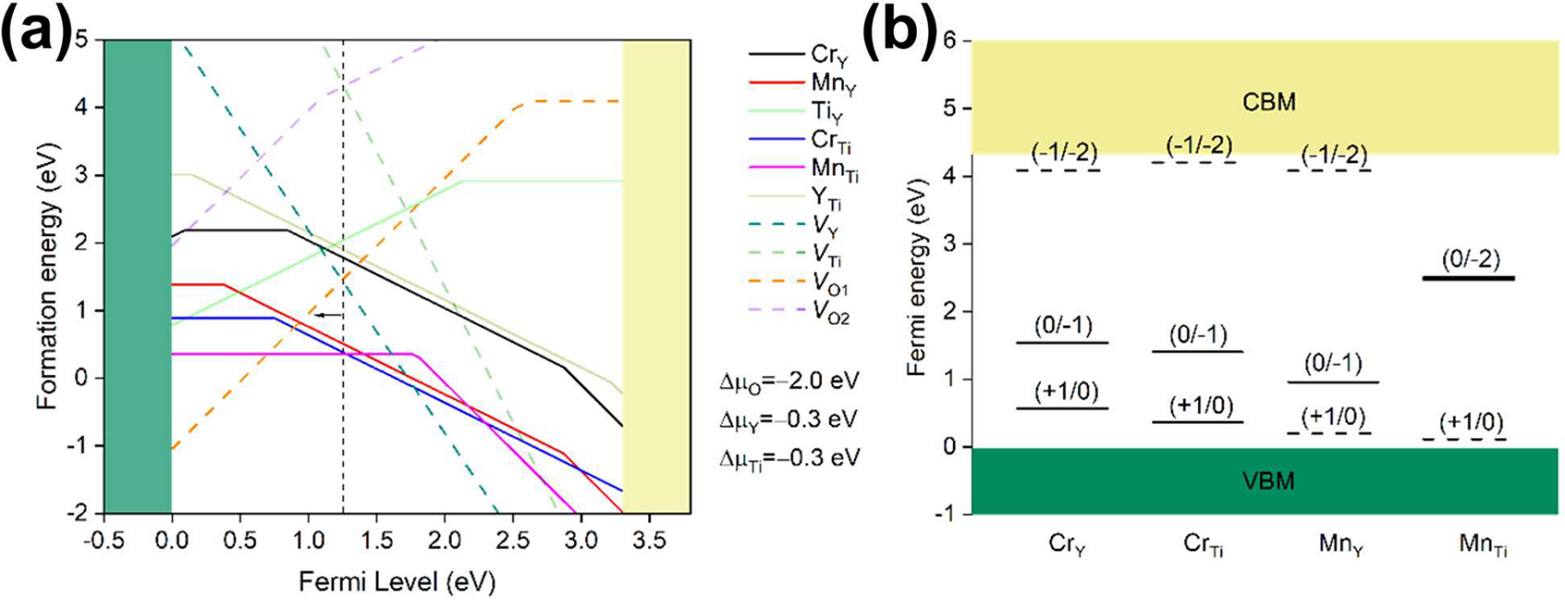
(a) The formation energies of the intrinsic, Cr-doped, and Mn-doped defects in Y_2_Ti_2_O_7_, where the dashed vertical line represents the Fermi level under charge equilibrium with negligible doping concentration. In the calculation of the formation energies, the atomic chemical potentials are chosen as −2.0 eV (O), −0.3 eV (Ti), −0.3 eV (Y), 0.0 eV (Cr), and 0.0 eV (Mn) by taking the usual phases of O_2_, TiO_2_, Y_2_O_3_, Cr_2_O_3_, and Mn_3_O_4_ as references. It is noted the actual formation energies of all the defects of the same dopant (Cr or Mn) can be shifted upward (but not downward) according to the doping concentration, as the Cr- or Mn-rich condition has been employed. (b) The charge transition levels of main Cr-doped and Mn-doped defects, which are calculated with the HSE06 functional. The dashed lines close to the band edge represent the polaron levels of the VBM and CBM.

Figure 7 plots the formation energies of defects and charge transition levels of dopants of interest in Y_2_Ti_2_O_7_, where a weak reduction condition of Δμ_O_ = −2.0 eV relative to the energy of the (1/2)O_2_ molecule and the equal distribution of the calculated relative enthalpy of formation of *H*(Y_2_Ti_2_O_7_) – *H*(Y_2_O_3_) – 2*H*(TiO_2_) = −1.2 eV onto Y and Ti, i.e., Δμ_Y_ = Δμ_Ti_ = −0.3 eV relative to [*H*(Y_2_O_3_) – 3Δμ_O_]/2 and *H*(TiO_2_) – 2Δμ_O_, is adopted.

As shown by Figure 7a, the main intrinsic defects are V_Y_^′′′^ and oxygen vacancies (*V*_O_^••^). The dashed vertical line represents the Fermi level under charge equilibrium under dilute limits of Cr and Mn dopants, and the Fermi level will move downward as the doped defect concentration increases due to charge neutrality constraints. Under dilute limits, the main doped defects are Cr_Ti_^′^ (Cr^3+^ occupying Ti^4+^ site) and Mn_Ti_^×^ (Mn^4+^ occupying Ti^4+^ site), which are followed by Mn_Y_^′^ (Mn^2+^ occupying Y^3+^ site), and their formation energies will, respectively, increase by 0, −2/3 μ, and +1/3 μ as Δμ_O_ increases by μ. Therefore, the more reducing atmosphere will favor the appearance of Mn_Y_^′^.

The charge transition level calculation with the HSE06 functional is shown in Figure 7b. The gaps Cr_Y_ (0/–1), Cr_Ti_ (0/–1), and Mn_Y_ (0/–1)Mn_Ti_ (0/–2) and CBM, VBM, and Mn_Ti_ (0/–2) are all about 2.5–3.0 eV, which may be involved in optical absorption and luminescence quenching processes.

## 3 Conclusions

The emission spectrum of our undoped sample of Y_2_Ti_2_O_7_ exhibits bands due to Cr^3+^ and Mn^4+^ impurities. The starting materials were 99.995% TiO_2_ and 99.999% Y_2_O_3_. However, the use of an Al_2_O_3_ crucible is known to introduce a Cr^3+^ impurity. The source of the Mn^4+^ impurity is not known. The spectrum of Cr-doped Y_2_Ti_2_O_7_ clearly shows that the previously reported Bi^3+^ emission^12^ in fact corresponds to Cr^3+^. The emission lifetime was reported as 17 μs.^12^ Our measurement at 288 K gives the value 13 μs. Analysis of the starting materials and the alumina crucible by inductively coupled plasma-mass spectrometry (ICP-MS) shows that the elements Cr, Mn, Fe, and Bi are present at ppb levels (Supporting Information).

The Cr^3+^ and Mn^4+^ emissions are quenched with an increasing temperature. In fact, the Mn^4+^ emission is barely visible above 80 K. We speculate about the possible processes involved. For Cr^3+^, its excited state may ionize to Cr^4+^, and the electron converts Ti^4+^ to Ti^3+^. On the other hand, the excited Mn^4+^ may ionize to form Mn^3+^ and a hole.

From our measurements, Mn-doped Y_2_Ti_2_O_7_ has the potential to function as a temperature sensor below 80 K.

## 4 Experimental Section

### 4.1 Materials

The yttrium(III) oxide (Y_2_O_3_, 99.999%), bismuth(III) oxide (Bi_2_O_3_, metal basis, 99.999%), manganese(IV) oxide (MnO_2_, metal basis, 99.996%), and chromium(III) oxide (Cr_2_O_3_, metal basis, 99.97%) were purchased from Alfa Aesar. The titanium(IV) oxide (TiO_2_, trace metal basis, 99.995%) was purchased from Sigma-Aldrich. The iron(III) oxide (Fe_2_O_3_, trace metal basis, 99.999%) was purchased from Acros Organics. The above chemicals were used without further purification.

### 4.2 Synthesis

A series of phosphors were prepared: undoped Y_2_Ti_2_O_7_, Y_2–*x*_Ti_2_O_7_: *x* mol % Bi^3+^ (*x* = 0.1, 0.5, 1, and 3), Y_2–*x*_Ti_2_O_7_: *x* mol % Fe^3+^ (*x* = 0.1, 0.5, 1, and 3), Y_2–*x*_Ti_2_O_7_: *x* mol % Mn^4+^ (*x* = 0.1 and 3), and Y_2–*x*_Ti_2_O_7_: *x* mol % Mn^3+^: *x* = 0.1 and 3). Phosphors were synthesized by a high-temperature solid-state reaction method. Y_2_O_3_, TiO_2_, Fe_2_O_3_, Bi_2_O_3_, MnO_2_, and Cr_2_O_3_ were weighed according to the stoichiometry of the chemical formula. Here, 2 mL of ethanol was added to the mixtures, and the mixtures were fully ground in an agate mortar until the ethanol was evaporated. The evenly mixed powder was then transferred to an aluminum crucible, treated at 1000 and 1600 °C for 4 and 10 h, respectively, with intermediate grinding. After regrinding, the final phosphors were obtained.

### 4.3 Characterization

The X-ray diffraction (XRD) patterns of samples were collected by a Bruker AXS D8 Advance X-ray diffractometer at 40 kV and 40 mA with Cu Kα radiation (λ = 1.5418 Å). The photoluminescence spectra at room and lower temperatures were recorded by a Horiba Fluorolog-3 spectrophotometer, using a 450 W xenon lamp as the light source. A 355 nm Spectra LED was employed to measure the decay curves, with a minimum range of 340 μs. A CS202-DMX-1AL cryostat from Advanced Research Systems (DE-202 series closed-cycle cryostat) was employed to record spectra in the nominal temperature range from 10 to 288 K. X-ray photoelectron spectra were obtained using a Thermo Scientific ESCALAB 250Xi instrument. ICP-MS (inductively coupled plasma–mass spectrometry) analysis was performed using an Agilent Technologies 7900 series ICP-MS instrument. The samples for ICP-MS measurements were prepared by dissolving 1 mg of Y_2_O_3_, TiO_2_, or alumina crucible in 1 mL of concentrated nitric acid (70%, trace metal basis) at 200 °C for 24 h. The resulting solution was diluted with milli-Q water to reach a total volume of 50 mL for measurements.

### 4.4 Calculations

The calculations were performed with the VASP code^44,45^ by employing the projected augmented wave method.^46^ The Perdew–Burke–Ernzerhof revised for solids (PBEsol) functional^47^ was chosen, and the cutoff energy is 520 eV. The single Γ-point sampling was adopted in the supercell calculations. The convergence criteria of energy and residual atomic forces are 10^–5^ and 10^–2^ eV Å^–1^, respectively. For the correction on the localization of *d* orbitals, the PBE+*U* method^48^ (*U* = 4 eV) is used in the formation energy calculation, and the Heyd–Scuseria–Ernzerhof hybrid (HSE06) functional^49^ with α = 0.25 is used in the charge transition level (CTL) calculation. More details about the calculation of formation energy and charge transition levels of a point defect and the correction have been given in previous work.^2^

## Data Availability

The research data are available upon reasonable request to the authors.

## Supporting Information Available

XPS and ICP-MS analysis results and emission spectra of Y_2_Ti_2_O_7_ doped with Fe (PDF)
