# Supplementary material for: Comment on “Charge Transfer-Triggered Bi3+ Near-Infrared Emission in Y2Ti2O7 for Dual-Mode Temperature Sensing”
Source: ACS Appl Mater Interfaces. 2023 Sep 11;15(37):43226–33. doi: 10.1021/acsami.3c11758 (PMC10772939; doi:10.1021/acsami.3c11758)
Supplement: Supplementary file 1 — am3c11758_si_001.pdf [file am3c11758_si_001.pdf]

Supporting Information

Comment on “Charge Transfer-Triggered Bi^{3+} Near-Infrared Emission in $Y_2Ti_2O_7$ for Dual-Mode Temperature Sensing”

Hei-Yui Kai[a], Longbing Shang[b,c], Ka-Leung Wong[d]*, Chang-Kui Duan[b,c]*, Peter A. Tanner[a]*

[a] Department of Chemistry, Hong Kong Baptist University, Waterloo Road, Kowloon Tong, Hong Kong S. A. R., P. R. China

[b] CAS Key Laboratory of Microscale Magnetic Resonance, and School of Physical Sciences, University of Science and Technology of China, Hefei 230026, P. R. China

[c] CAS Center for Excellence in Quantum Information and Quantum Physics, University of Science and Technology of China, Hefei 230026, P. R. China

[d] Department of Applied Biology and Chemical Technology, The Hong Kong Polytechnic University, Hung Hom, Kowloon. Hong Kong S. A. R., P. R. China

Corresponding author*: klgwong@polyu.edu.hk ; ckduan@ustc.edu.cn; peter.a.tanner@gmail.com

Keywords: Mn^{4+} emission, Cr^{3+} emission, Fe^{3+} emission, impurity emission, pyrochlore, Bi^{3+} doping

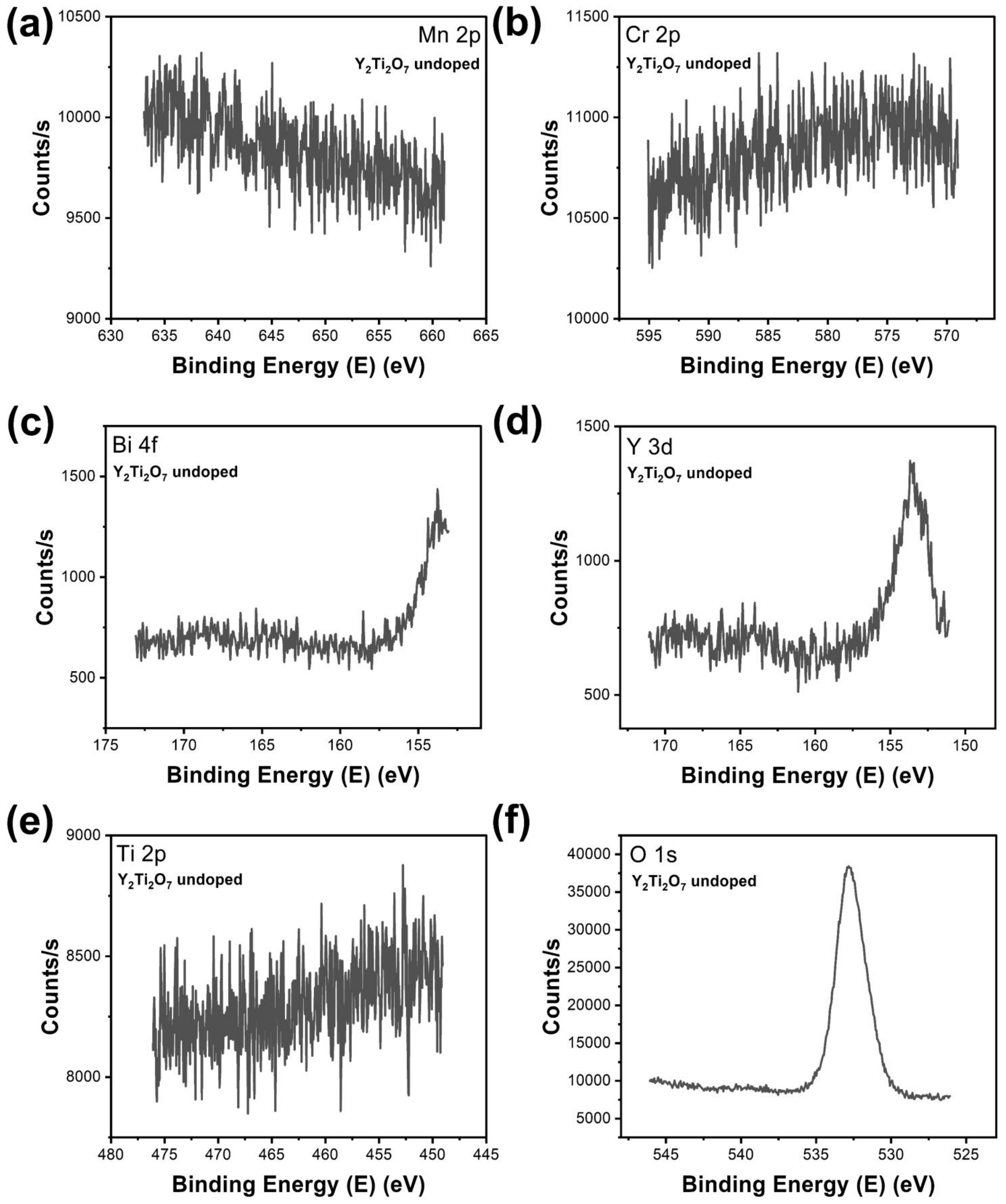

Figure S1. High resolution XPS spectra of undoped $Y_2Ti_2O_7$: (a) Mn 2p; (b) Cr 2p; (c) Bi 4f; (d) Y 3d; (e) Ti 2p; (f) O 1s.

Table S1. Result of Y_2O_3, TiO_2, and alumina crucible analyses by ICP-MS.

Sample	Concentration (ppb)			
	52 Cr	55 Mn	56 Fe	209 Bi
Alumina crucible	26.1±0.4	9.9±0.4	124±19	8.7±0.4
TiO_2	51.1±1.9	17.2±1.1	100±14	24.9±0.7
Y_2O_3	18.9±0.6	2.8±0.4	90±2	6.3±0.1

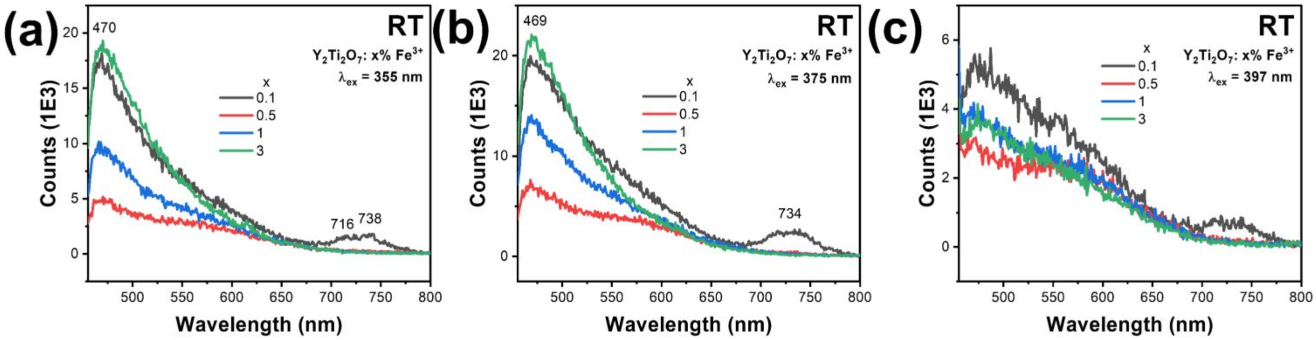

Figure S2. Emission spectra of 0.1% Fe-doped $Y_2Ti_2O_7$ under (a) 355 nm; (b) 375 nm; (c) 395 nm excitation.
